# Pelger‐Huët anomaly and T‐cell lymphoma

**DOI:** 10.1002/jha2.184

**Published:** 2021-03-21

**Authors:** Roland Schroers, Thomas Mika

**Affiliations:** ^1^ Department of Medicine Hematology and Oncology Ruhr‐University Bochum Bochum Germany

**Keywords:** Pelger‐Huët‐anomaly, T‐cell lymphoma


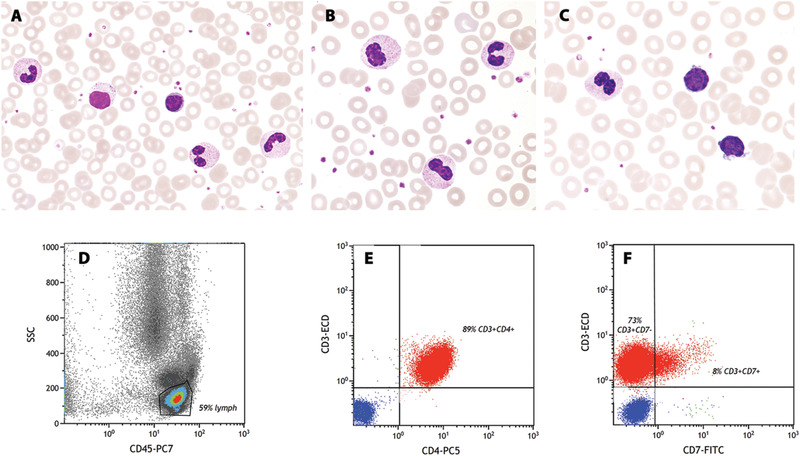


A 69‐year‐old man was referred for evaluation of general lymphadenopathy without B‐symptoms and without skin rash. Peripheral blood parameters were 140 g/L of haemoglobin, platelets 167 × 10^9^/L and leukocytes 67 × 10^9^/L. The lactate dehydrogenase (LDH) concentration was markedly elevated (1467 U/L). Differential blood counts were 45% neutrophils, 50% lymphocytes, 4% monocytes and 1% eosinophils. Blood smears showed neutrophils with dense clumped nuclear chromatin indicating maturity (panel A, May‐Grünwald stain, 63x objective). However, all neutrophils lacked nuclear segmentation and had rod shaped, rounded or bilobed nuclear contours (panel B, 100x objective). These features were characteristics of Pelger‐Huët‐anomaly. Furthermore, several atypical lymphocytes with large cytoplasmic granules (panel A, 63x objective), cytoplasmic protrusions and indented nuclei (panel C, 100x objective) were observed. In flow cytometry and immunophenotyping, the majority were mature lymphocytes with expression of CD3, CD4 and CD5, but partial loss of CD7 (panels C, E, D; density and dot plots). Bone marrow pathology and T‐cell receptor clonality ascertained the diagnosis of a leukemic variant of T‐cell lymphoma.

Pelger‐Huët‐anomaly is an autosomal dominant haematological trait caused by lamin B receptor mutations. Equivalent nuclear hypolobulation and chromatin aberration in neutrophils are associated with various haematological malignancies such as myelodysplastic and lymphoproliferative disorders and also non‐Hodgkin's lymphoma. In the reported case, Pelger‐Huët‐anomaly together with atypical lymphocytes gave an important hint towards the diagnosis.

